# “De Novo” Psoriasis and Relapse of Psoriasis Induced by Dupilumab: Three New Cases and Review of the Literature

**DOI:** 10.3390/jcm12196291

**Published:** 2023-09-29

**Authors:** Ilaria Trave, Ilaria Salvi, Martina Burlando, Emanuele Cozzani, Aurora Parodi

**Affiliations:** Section of Dermatology, DISSAL, University of Genoa, IRCCS Ospedale Policlinico San Martino, 16044 Genova, Italy

**Keywords:** atopic dermatitis, psoriasis, treatment, biologics, target therapies

## Abstract

Atopic dermatitis and psoriasis are traditionally considered diseases that cannot coexist, since they are described as the result of the activation of opposing inflammatory pathways. However, this belief has been debunked, and numerous cases of psoriasis induced by dupilumab, a biologic treatment for atopic dermatitis, have been reported. We report three cases of dupilumab-induced psoriasis and we present a literature review including cases of “de novo” psoriasis and of the relapse of psoriasis that occurred during treatment with dupilumab. In total, 39 publications met the inclusion criteria, including 112 AD patients, 101 of whom developed “de novo” psoriasis, and 11 with a flare of pre-existent psoriasis. In the first group, patients more frequently developed plaque psoriasis on the scalp and extremities, after an average latency period from the initiation of dupilumab of 5 months. In the second group, the incidence of dupilumab-induced relapses of psoriasis was 43%, after an average of 4 months since the first administration. The most common psoriasis type was plaque psoriasis, with the involvement of the scalp and upper extremities. Dupilumab was interrupted in 38% of patients with “de novo” psoriasis and in 50% of relapsed patients, leading, in most cases, to an improvement of psoriasis. In conclusion, atopic dermatitis and psoriasis can definitely co-exist, and biologic drugs used to treat the former can promote the latter. It is thus crucial to perform a careful personal and familiar anamnesis before prescribing any biologic treatment. Moreover, a study of cytokine expression and blood proteomic markers could be considered in these patients.

## 1. Introduction

In the literature, atopic dermatitis (AD) and psoriasis (PsO) are described as two diseases that cannot coexist in the same patient due to the activation of opposing inflammatory pathways in these illnesses, in particular T helper 2 (TH-2) in AD and T helper 1 (TH-1) in PsO [1]. Nonetheless, cases and systematic reviews have also reported that AD and PsO may coexist in the same patient or subsequently develop due to different causes. For example, the use of dupilumab for AD may trigger a flare of PsO in patients with a previous history of this illness or induce “de novo” PsO in some AD patients who claimed no history of PsO. Dupilumab is a fully human IgG4 antibody directed against the interleukin (IL)-4 receptor alpha chain, thus blocking IL-4 and IL-13 signaling, modulating the T helper (Th)2-mediated inflammation. Both IL-4 and IL-13 are known to be able to downregulate IL-23 from antigen-presenting cells or IL-17 from T cells [2]. In clinical trials, conjunctivitis, herpes infections and injection-site reactions were found to be the most frequently observed side-effects [3]. Paradoxical erythema distributed in the head and neck area, arthritis, alopecia areata and PsO were other mild collateral effects reported in the long-term surveillance [4]. In 2018, Tracey et al. [5] reported the first case of erythrodermic PsO associated with treatment with dupilumab. Since then, numerous cases of dupilumab-associated “de novo” PsO and relapse of PsO in AD patients have been reported.

In this study, we have described three cases of “de novo” PsO and relapse of PsO developed in AD patients treated with dupilumab and we present a review of the literature of case reports of this adverse reaction. 

## 2. Materials and Methods

### 2.1. Patients

All patients with “de novo” PsO and relapse of PsO visited in Dermatology Clinic, IRCCS, Ospedale Policlinico San Martino, Genoa, Italy were included in the study.

### 2.2. Literature Search

We performed a Pubmed search including articles from January 2018 to May 2023 using the following search terms: “psoriasis”, “psoriatic”, “psoriasiform” and “dupilumab”. In addition, articles including the wording “case report” or “case series” were added. All the sourced articles were full-text reviewed to ensure that the contents were relevant to the study. 

## 3. Results

### 3.1. Patients

**Figure 1:**
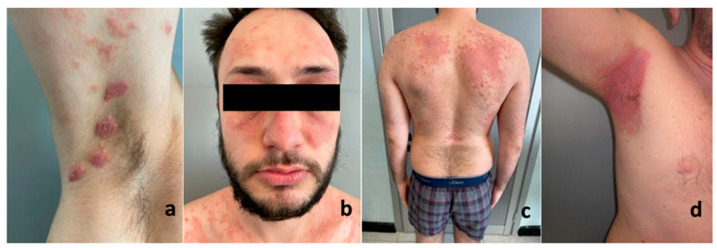
Psoriasis on the upper lower extremities in case 1 (**a**). Face atopic dermatitis (**b**) and trunk psoriasis (**c**) on case 2. Inverse psoriasis in case 3 (**d**).

Case 1 (Figure 1a). A 16-year-old boy, with neither a family nor a personal history of PsO, presented with severe childhood-onset AD, which was recalcitrant to topical and systemic corticosteroids. In anamnesis, he denied any previous history of asthma or allergic rhinitis. His Eczema Area and Severity Index (EASI) score was 30. Hence, after a loading dose of 600 mg, dupilumab, 300 mg subcutaneously was started. The patient experienced rapid improvement in his AD. However, after 4 weeks, he developed PsO plaques on his arms and neck associated with erythema and the scaling of the face and scalp with a Psoriasis Area and Severity Index (PASI) score of 2.5 (Figure 1a). Topical clobetasol was started and dupilumab was continued until 12 weeks later, when both his AD and PsO worsened (EASI 40, PASI 5.2). The patient declined a biopsy of the skin lesions. Dupilumab was then discontinued and ciclosporin, 3 mg/kg/day, was started. Within 4 weeks, the patient experienced the disappearance of his body and face PsO and his AD improved.

Case 2. A 25-year-old man, with a family and personal history of PsO, presented with a severe childhood-onset AD of 15-year duration, which was recalcitrant to systemic corticosteroids and 3 mg/kg/day cyclosporine. A single episode of plaque PsO was reported on his elbows 10 years ago. The patient reported a worsening of oculorhinitis and AD on the eyelids over the last 4 years. The last patch test resulted negative and excluded a simultaneous contact dermatitis. His EASI score was 35 and PsO lesions were absent. Thus, after a loading dose of 600 mg, dupilumab, 300 mg subcutaneously was started, while cyclosporine and prednisolone were tapered and stopped. The patient experienced rapid improvement in his AD. However, after 4 weeks he developed PsO plaques on the arms and trunk. His PASI score was 4.5. Topical clobetasol was started and dupilumab was continued until 4 weeks later, when both his AD and PsO worsened (Figure 1b,c). The patient declined a PsO plaque biopsy and dupilumab was then discontinued and instead methotrexate (12.5 mg once a week plus one 5 mg tablet of folic acid the following day) was started. Within 4 weeks, the patient experienced partial AD and PsO improvement (EASI 10, PASI 2). After 3 months, PsO was completed resolved. 

Case 3. A 25-year-old man had been suffering from moderate–severe AD in the last 5 years. At first, he was treated with topical steroids and H1-antihistamines, later replaced with 300 mg/die of cyclosporine. Two years ago, he was diagnosed with ulcerative colitis and began a treatment with prednisone 5 mg/die and mesalazine 800 mg × 2/die. After the worsening of AD in the last year, he started treatment with dupilumab (an initial loading dose of 600 mg, followed by 300 mg every two weeks). Consequently, cyclosporine was progressively tapered; the AD rapidly improved but he developed PsO with a PASI of 18 (Figure 1d) and consistent patchy hair loss with alopecia areata after 8 weeks. Dupilumab was immediately interrupted, prednisone was increased from 5 mg to 25 mg/die and 300 mg/die of cyclosporine was reintroduced, along with topical clobetasol propionate 0.05% scalp application. After 3 months, hair regrowth was complete and, in agreement with the patient’s gastroenterologist, prednisone was definitely withdrawn and infliximab, a biologic drug indicated for both PsO and ulcerative colitis, was started with a dosage of 5 mg/kg at time 0, after 2 weeks, then after 4 weeks and later every 8 weeks. The PsO soon improved reaching a PASI of 2, but the patient suffered a rebound of AD, which was managed with topical treatments. In addition, infliximab proved ineffective on the gastroenterological disease, so we decided to start ustekinumab. We chose to treat this patient with the gastrenterological dosage (a single IV induction dose of 520 mg, followed by 90 mg subQ every 8 weeks). The patient greatly improved from the dermatologic and gastroenterological diseases and is currently in remission.

### 3.2. Literature Search (Table 1, Table 2, Table 3 and Table 4)

In total, 39 publications met the inclusion criteria, including 112 AD patients, and in particular, 101 patients who developed “de novo” PsO (93 adult patients and 8 pediatric patients) and 11 patients with a flare of pre-existent PsO (10 adult patients and 1 pediatric patient) developed after dupilumab treatment. Dupilumab was prescribed for AD in 102 patients, for “nonspecific” dermatitis in 4 patients, for prurigo nodularis in 1 patient and for nasal polyposis in 4 patients. The average age of patients affected by “de novo” PsO and pre-existent PsO flare was, respectively, 43 years (range, 4–80 years old) and 26 years (range, 9–50 years old) in the adult group, respectively. The average time from dupilumab initiation to “de novo” PsO onset was 5.6 months (range 1–30 months) in adults. The time between start of treatment and “de novo” PsO onset was slighty longer in pediatric patients than in adults (6.4 months, range 2–10 months). On the other hand, the flare of pre-existent PsO presented a relatively shorter time to onset of 4.8 months on average in both adult and pediatric patients. The “de novo” PsO was characterized by plaque PsO in 42/101 patients, namely in 34/93 adults and 8/8 pediatric patients; pustular psoriasis in 8/93 adults and 1/8 pediatric patients; erythrodermic psoriasis in 3/93 of adults; guttate PsO in 3/93 adult patients; reverse PsO in 2/93 of adult patients and nummular psoriasis in only 1 adult. The lesions of “de novo” PsO were more frequently localized at the scalp (12/93 of adults and 3/8 of pediatric patients) and simultaneously at the superior and inferior extremities (11/93 of adults and 2/8 of pediatric patients). Patients with a flare of pre-existent PsO more frequently presented plaque PsO (9/10 of adults and 1 pediatric patient), erythrodermic PsO (3/10 of adults) and guttate PsO (1/10 of adults). Lesions were more frequently localized at the upper extremities (6/10 in adults and 1 pediatric patient) and at the scalp (5/10 in adults).

**Table 1:** Case reports and case series of “de novo” PsO in adults.

References	N° Cases	Age and Gender (Years)	Duration, (Month)	Type	Localization	Histology	Dupilumab Interruption	Management	Outcome
Safa G, 2019 [6]	1	55, M	2	Plaque	Trunk	Yes	No	Topical steroids	Improvement
Fowler E, 2019 [7]	2	54, F	4	Plaque	Upper and lower extremities, chest, back, neck, abdomen, soles and palms	Yes	Yes	NA	Improvement
	/	49, F	18	Plaque	Bilateral upper and lower extremities, nails	NA	No	Topical steroids	Improvement
Stout M, 2019 [8]	1	59, F	1	Plaque	Bilateral upper and lower extremities	Yes	Yes	Topical steroids	Improvement
Gori N, 2019 [9]	1	40, F	3.5	Guttate	Trunk and extremities	Yes	No	Topical calcipotriol and steroid	Improvement
Varma A, 2020 [10]	1	73, M	1	Plaque	Bilateral upper extremities and right lower extremities	NA	NA	NA	NA
Schrom KP, 2020 [11]	1	80, M	2.5	Plaque	Trunk, upper extremities and lower extremities	Yes	Yes	NB-UVB	Improvement
Ferrucci SM, 2020 [12]	1	42, M	3	Plaque	Trunk and lower extremities	Yes	Yes	Topical calcipotriol and steroids	Improvement
Kim HS, 2020 [13]	1	36, F	5	Plaque	Lower extremities	Yes	Yes	Topical steroids	Recurrence
Gambichler T, 2020 [14]	1	59, M	1	Erythrodermic	Upper and lower extremities	Yes	Yes	NA	NA
Matsuda T, 2020 [15]	1	60, M	3.5	Plaque	Upper and lower extremities, face	Yes	Yes	Topical steroids	Improvement
DeGrazia TM, 2020 [16]	3	32, F	1	Plaque	Scalp, bilateral inguinal folds	NA	No	Topical steroids	NA
	/	67, M	2	Plaque	Scalp	NA	No	Topical steroids	NA
	/	57, F	9	Plaque	Scalp	NA	No	Topical steroids	NA
D’ambra I, 2020 [17]	3	61, F	1	Guttate	Upper and lower extremities and trunk	NA	No	Topical steroids	Improvement
	/	56, F	2	Plaque	Upper extremities and scalp	Yes	No	Topical calcipotriol and steroids	Improvement
	/	39, M	1	Plaque	Soles	NA	No	Topical steroids	Improvement
Senner S, 2020 [18]	1	40, M	1.5	Plaque	Lower extremities and palms	Yes	Yes	Topical steroids, oral steroids, cyclosporine	Improvement
Al-Janabi A, 2020 [19]	1	72, M	4	Seborrheic	Scalp, face and ears	NA	No	Topical steroids	Improvement
Gallo R, 2020 [20]	1	24, M	1.5	Reverse	Bilateral inguinal folds and scalp	No	Yes	Topical steroids and cyclosporine	Improvement
Mirza FN, 2021 [21]	1	92, M	8	Plaque	Upper and lower extremities	Yes	No	Mycophenolate mofetil	Improvement
Beaziz J, 2021 [22]	1	45, F	12	Plaque	Scalp	NA	No	Topical steroids	Improvement
Maiolini VM, 2021 [23]	1	22, M	5	Plaque	Scalp, left upper extremity, nails	Yes	NA	Topical calcipotriol and steroid	Improvement
Russo F, 2021 [24]	1	68, F	1	Plaque	Upper extremities and buttocks	Yes	Yes	Oral steroids	Improvement
Kurihara K, 2021 [25]	2	34, M	30	Plaque	Upper extremities and trunk	Yes	No	Topical steroids, tacrolimus ointment 0.1%, delgocitinib ointment	Improvement
	/	23, M	18	Plaque	Face	Yes	No	Topical steroids, tacrolimus ointment 0.1%, delgocitinib ointment	Improvement
Park J, 2021 [26]	1	24, M	2	Plaque	Palms and soles	Yes	No	Topical steroids	Improvement
Juan-Carpena G, 2021 [27]	1	46, M	24	Erythrodermic	Lower extremities and trunk	Yes	No	Topical steroids, tacrolimus ointment 0.1%	Persistence
Flanagan KE, 2022 [28]	1	28, F	5	Plaque	Scalp and abdomen	NA	Yes	Topical steroids	Improvement
Fan J, 2022 [29]	1	25, F	2	Inverse	Armpits	NA	Yes	Topical hormonal cream	Improvement
Incel Uysal P, 2022 [30]	1	22, M	4	Pustular	Hands	Yes	Yes	Topical calcipotriol and steroids	Improvement
Jia X, 2022 [31]	1	23, M	2	Pustular	Lower extremities	Yes	Yes	Topical steroids, cyclosporine	Improvement
Patruno C, 2022 [32]	1	58, F	5.5	Plaque	Lower extremities, scalp, hands	Yes	Yes	Upadacitinib	Improvement
Zhong X, 2022 [33]	1	51, F	2	Pustular	Upper extremities and trunk	Yes	Yes	Oral steroids	Improvement
Paolino G, 2022 [34]	4	36, M	25	NA	NA	NA	No	Topical calcipotriol and steroids	Improvement
	/	36, F	25	NA	NA	NA	No	Topical calcipotriol and steroids	Improvement
	/	36, F	25	NA	NA	NA	No	Topical calcipotriol and steroids	Improvement
	/	36, F	25	NA	NA	NA	No	Topical calcipotriol and steroids	Improvement
Casale F, 2022 [35]	4	61, M	10.5	Plaque	Inferior extremities	Yes	Yes	Topical steroids	NA
	/	61, M	10.5	Plaque	Scalp	No	Yes	Topical steroids	NA
	/	61, M	10.5	Plaque	Upper extremities	No	No	Topical steroids, cyclosporine	NA
	/	61, F	10.5	Erythrodermic	NA	No	No	Cyclosporine	NA
Grolleau C, 2023 [36]	7	20, F	1	Plaque	NA	Yes	Yes	Topical steroids, baricitinib	Partial
	/	50, M	4	Pustular	NA	Yes	Yes	Topical steroids, methotrexate	Partial
	/	25, F	0.5	Plaque, pustular	NA	Yes	Yes	Upadacitinib	NA
	/	47, M	6.5	Plaque, pustular	NA	Yes	Yes	Upadacitinib	NA
	/	39, M	10	Plaque	NA	Yes	No	NA	Partial
	/	84, M	1.5	Plaque	NA	Yes	Yes	Methotrexate	Improvement
	/	65, M	2.5	Pustular	NA	Yes	Yes	Methotrexate	Partial
Napolitano M, 2023 [4]	39	54, M (26) F (13)	5	NA	NA	Yes	Yes (10), No (29)	Topical steroids (26), NB-UVB (11), methotrexate (2)	Improvement (29), Worsening (6), Persistence (4)
Al Hawsawi K, 2023 [37]	1	50, F	1	Plaque	Scalp	NA	Yes	Topical steroids	Recurrence
Chromy D, 2023 [38]	4	30, M	1	Nummular	Upper and lower extremities	Yes	No	Topical calcipotriol and steroids	Recurrence
	/	48, F	3.5	Plaque	Upper and lower extremities, trunk, scalp, face	Yes	Yes	Topical calcipotriol and steroids and omalizumab	Improvement
	/	36, M	4.5	Pustular	Face	No	No	NA	NA
	/	82, M	6	Guttate	NA	Yes	No	Topical steroids	Improvement
Our case 3	1	25, M	8	Inverse	Armpits and scalp	No	Yes	Topical and systemic steroids and ciclosporine	Improvement

**Table 2:** Case reports and case series of “de novo” PsO in pediatric patients.

References	N° Cases	Age and Gender (Years)	Duration, (Month)	Type	Localization	Histology	Dupilumab Interruption	Management	Outcome
Parker JJ, 2021 [39]	4	4, M	11	Plaque and pustular	Lower extremities and trunk	No	Yes	Topical steroids, ustekinumab	Improvement
	/	14, F	8	Plaque	Face	No	No	Topical steroids	Improvement
	/	12, F	10	Plaque	Lower extremities	No	No	Topical steroids	Improvement
	/	16, M	7	Plaque	Lower extremities	No	No	Topical steroids	Improvement
Park J, 2021 [26]	1	17, M	2	Plaque	Palms and soles	Yes	No	Topical steroids, tacrolimus ointment 0.1%	Improvement
Ali K, 2022 [40]	2	17, M	5	Plaque	Upper and lower extremities, back, face, scalp	Yes	Yes	Baricitinib	Improvement
	/	17, M	5	Plaque	Upper and lower extremities, back, face, scalp	Yes	Yes	Baricitinib	Improvement
Colonna C, 2022 [41]	1	9, M	3	Plaque	Upper extremities, trunk	Yes	NA	Topical steroids	Improvement
Our case 1	1	16, M	1	Plaque	Upper extremities, face and scalp	No	Yes	Topical steroids and cyclosporine	Improvement

**Table 3:** Case reports and case series of relapse of PsO in adults.

References	N° Cases	Age and Gender (Years)	Duration, (Month)	Type	Localization	Histology	Dupilumab Interruption	Management	Outcome
Tracey EH, 2018 [42]	1	50, F	2	Erythrodermic	Upper and lower extremities, trunk and scalp	Yes	Yes	Topical steroids, methotrexate	Improvement
Dimitrov D, 2020 [42]	1	35, M	5	Plaque	Upper and lower extremities	Yes	NA	NA	NA
Parker JJ, 2021 [39]	2	18, M	7	Plaque	Scalp and face	NA	No	Topical steroids	Improvement
	/	18, F	6	Plaque	Upper extremities, scalp and trunk	NA	No	Topical steroids and pimecrolimus 1% cream	Improvement
Casale F, 2022 [35]	6	66, M (1), F (5)	5	Erythrodermic (2), plaque (5), guttate (1)	Scalp (2), face (1), upper extremities (5), inferior extremities (2), trunk (1), soles and palms (1)	Yes	Yes (2), No (4)	Topical steroids (6) and omalizumab (5)	NA
Our case 2	1	25, M	1	Plaque	Upper extremities, trunk	No	Yes	Topical steroids and methorexate	Improvement

**Table 4:** Case reports and case series of relapse of PsO in pediatric patients.

References	N° Cases	Age and Gender (Years)	Duration, (Month)	Type	Localization	Histology	Dupilumab Interruption	Management	Outcome
Parker JJ, 2021 [39]	1	9, F	3	Plaque	Upper extremities	No	No	Topical steroids	Improvement

A skin biopsy of “de novo” PsO was performed on 31/93 adults and on 4/8 pediatric patients. A biopsy of flare of PsO was performed on 3/10 adults. The histopathological features of “de novo” PsO in adult and pediatric patients and flare of PsO are shown in Table 5. 

Dupilumab was discontinued by 36/93 adult patients and 3/8 pediatric patients who developed “de novo” PsO and by 3/10 adult patients with a flare of PsO. 

Among 101 patients with “de novo” PsO, 63/93 adults and all 8/8 pediatric patients achieved a complete remission following dupilumab discontinuation (26/63 adults and 4/8 pediatric patients) and PsO treatments. In particular, among 101 patients with “de novo” PsO, topical steroids were the most frequently used treatments (53/93 adults and 7/8 pediatric patients) followed by NB-UVB (12/93 adults), an association between topical calcipotriol and steroid (11/93 adults), methotrexate (5/93 adults), cyclosporin (5/93 adults and 1 pediatric patient), oral steroids (3/93 adults), tacrolimus (3/93 adults and 1 pediatric patient), omalizumab (1/93 adults), mycophenolate mofetil (1/93 adults) and JAKi (upadacitinib in 2 adult patients, delgocitinib in 2 adult patients and baricitinib in 1 adult and in 2 pediatric patients). Among 11 patients with relapse of PsO, the most frequently administered treatments were topical steroids (5/11), omalizumab (5/11), methotrexate (1/11) and pimecrolimus 1% cream (1/11). Unfortunately, among cases of “de novo” PsO, 3 patients had an incomplete response from the suspension of dupilumab, 1 patient reported a recurrence of PsO during the time, 5 patients reported a persistence of PsO and 6 patients a worsening of PsO.

Main findings from the reviewed and new cases were showed in Table 6.

## 4. Discussion

After performing a review of the literature, we found that two different groups of AD patients have been observed developing PsO during dupilumab treatment: the group of “de novo” PsO patients and those with flare ups of pre-existent PsO. In our review, we chose to divide these two groups, since we believe that the onset of a new disease and the relapse of pre-existing PsO are different entities, from both a pathogenetic and a clinical perspective.

We found that a greater number of cases of “de novo” PsO has been reported, while the relapse of pre-existing disease seemed less frequent. This reflects our own experience since we had only one case of relapse of PsO.

In the first group, patients more frequently developed new PsO plaques on the scalp and upper and lower extremities after an average latency period from initiating dupilumab to the onset of PsO of 5 months. Scalp lesions were common in both the “de novo” and relapse groups, which is unsurprising considering that this is generally one of the most frequently affected areas. Another frequent feature was pustular PsO at the upper and lower extremities, while erythrodermic and inverse PsO were less frequently reported. The prevalence of pustular psoriasis was higher in the “de novo” group, however more data would be needed to confirm and hypothesize an explanation for this finding. The incidence of “de novo” PsO in these patients is similar to the prevalence of classic PsO in the general population, namely 1.88% [43]–3.33% [44]. Two out of three of our reported AD patients developed “de novo” PsO following the dupilumab treatment. The onset of PsO was more rapid in our cases than in those reported in the literature. This finding could in fact be explained by an earlier diagnosis due to our attention to this phenomenon thanks to the numerous cases reported in the previous years. Atopic lesions remained stable or transitioned to a clinical appearance closer to PsO compared with the start of dupilumab. Plaque PsO appeared at the scalp in both of the patients and one of them also presented inverse PsO. 

The exact immunologic mechanism by which dupilumab induces the development of PsO in certain patients is not well known. Since the development of targeted therapies in the last few years, major studies attempting to portray a disease profile have been conducted, discovering different kinds of endotypes in patients affected by AD; endotypes are defined as the molecular mechanisms that can control the visible phenotype of AD [45,46]. For example, intrinsic AD, which is characterized by normal IgE levels, female predominance, delayed disease onset, higher metal contact hypersensitivity, and a lack of any other atopic background, usually presents an increased T helper 1 (TH-1) expression and a more pronounced TH-17/TH-22 activation [44]. Differently, extrinsic AD is characterized by high total IgE levels, eosinophilia, a personal or family atopic background and a greater rate of filaggrin gene mutations, and its endotype classically includes TH-2-TH-22 and TH-17 markers [47]. Moreover, different AD endotypes are found in different ethnic backgrounds. For example, an Asian AD endotype shows an increased expression of TH-17-TH-22; Caucasian–American AD reports more TH-2 [48], TH-22 biomarkers in the acute phase and TH-1 biomarkers in the chronic phase and African–American AD has a greater expression of TH-2-TH-22 with an absence of TH-17-TH-1 expression [49]. The expression of endotypes also changes with age; children have a very similar cytokines-expression to Asian AD with an overexpression of TH-22-TH-17 but an absence of TH-1-related cytokines [47]. 

When dupilumab inhibits the IL-4-IL-13 axis, these cytokines are no longer able to block the TH-17 pathway, which increases the IL-17-driven inflammation, which has been historically linked to PsO [50]. Napolitano et al. have demonstrated that the lesions of “de novo” PsO in patients with AD have a significant increase in IL-23A levels thus suggesting the activation of the TH-17 pathway [44]. IL-23A is a heterodimer proinflammatory cytokine mainly secreted by activated macrophages and dendritic cells. Another cytokine involved in the pathogenesis of “de novo” PsO may be IL-36, which is a surrogate marker of pustular PsO [36]. To sum up, dupilumab may promote an increase in PsO inflammation, which is, in our opinion, more critical in AD patients with a more pronounced expression of TH17-related cytokines, such as children. In our review, we found 8 AD children with “de novo” PsO; they developed more PsO plaques distributed at the scalp. However, the characteristics of patients who develop a psoriasiform reaction are still not clear.

In the second group, patients reported a personal history of PsO associated with AD. As reported in the literature, the incidence of relapses of PsO in these patients is 43% [51]. In our review, these patients reported a quicker development of PsO relapse, after 4 months from the start of the treatment and, clinically, they more frequently developed PsO plaques at scalp and upper extremities. Only one case of a pediatric patient with a reactivation of PsO was reported in the literature. 

To better understand the overlap phenotype between PsO and AD, the gene expression profiles of lesions and the proteomic profiles of blood samples of these patients were studied. The results were that patients with an overlap of AD and PsO showed a dominant genomic profile characteristic of classic PsO, with an overexpression of TH-17- and TH-1-regulated cytokines, different from the TH-2-regulated cytokine profiles of classic AD [52]. In addition, a RT-PCR study showed that the expression of TH-17 and TH-1 cytokines in the overlap phenotype are very similar to classic PsO. Moreover, the levels of blood protein biomarkers of PsO PI3, GDF 15, and TRAIL R2 were increased in the overlap phenotype as in PsO [52]. This study demonstrated that the overlap phenotype between PsO and AD is more similar to classical PsO than AD. Consequently, the acknowledgment of these overlap patients before the start of biologic treatment may be useful for a correct treatment of the patient.

In our review, a skin biopsy of “de novo” PsO was performed on 31/93 adults and on 4/8 pediatric patients. A biopsy of flare of PsO was performed on 3/10 adults. The two groups presented similar hystological features which were not always consistent with classic PsO but showed an overlap of psoriasiform and eczematous features [14]. Indeed, “de novo” and relapse of PsO presented not only in classic PsO histopathological features such as parakeratosis, hyperkeratosis, acanthosis of the epidermis, dilated capillaries, and dermal lymphocytic infiltration, but also mild spongiosis, which is a typical feature of AD. Differentiation between PsO and AD lesions in these patients can thus sometimes be difficult, both clinically and histologically. A genetic or cytokine study may be useful for distinguishing patients before the treatment. 

Regarding the management of “de novo” PsO and relapses of PsO in AD patients during treatment with dupilumab, no high-quality evidence-based management guidelines are currently available. It is common practice to treat mild cases with topical corticosteroids, without the discontinuation of dupilumab. The current literature suggests that topical steroids are particularly effective in pediatric patients with psoriasiform lesions, while adults tend to be more refractory to such treatment [39]. In moderate cases of “de novo” PsO, it may be appropriate to add phototherapy (narrow-band UVB) [44] and systemic therapies such as oral steroids, cyclosporine and methotrexate [44].

For severe cases, the discontinuation of dupilumab is recommended. In our review, 38% of adult patients and 38% of pediatric patients stopped dupilumab with complete remission and without a relapse of PsO. In cases of failure of the proposed treatments or in cases of contraindications to traditional systemic treatments, the combination of dupilumab and other monoclonal antibodies is another promising therapy strategy. For example, a biologic combination therapy with dupilumab and guselkumab has resulted in significant improvement in both AD and PsO [53]. However, long-term monitoring is necessary to evaluate the safety and the cost of these two combined biological treatments used together. 

**Figure 2:**
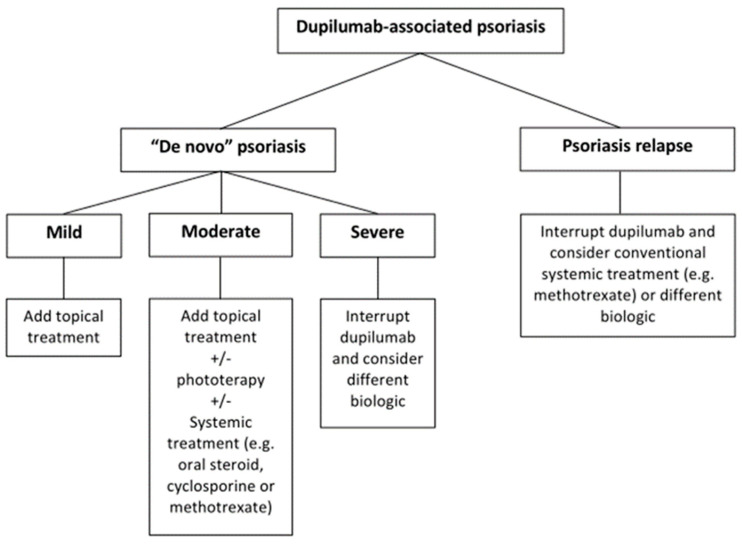
Proposed treatment algorithm for dupilumab-induced psoriasis.

Regarding the treatment of a flare of PsO in AD, we advise to immediately interrupt dupilumab because of the higher probability of PsO cytokines’ pattern of expression in these patients. For this reason, a drug which is effective on both PsO and AD should be more suitable for this form of overlap dermatitis (Figure 2). For example, in our experience, the only case of a patient with a relapse of PsO was treated with methotrexate with effectiveness on AD and PsO. Janus Kinase inhibitors (JAK inhibitors), recently approved for atopic dermatitis and psoriatic arthritis, have shown promising results in dupilumab-associated psoriasis. These drugs, belonging to the class of “small molecules”, reversibly inhibit JAK proteins, a family of receptor-associated kinases which participates to the “JAK-STAT signaling pathway”. Different JAKs are involved in the signal transduction of numerous cytokines and growth factors, including IL-4 and IL-13, the main mediators of atopic dermatitis [54]. The larger spectrum of activity of JAK inhibitors could justify their effectiveness in cases of overlapping features of psoriasis and atopic dermatitis. In particular, baricitinib [36,40] and upadacitinib [32,36] have been successfully used in these cases and delgocitinib ointment has been reported as effective [25]. However, the efficacy of these molecules in dupilumab-induced psoriasis has not been systematically investigated yet.

The limitations of our study include the high clinical heterogeneity of the data that were investigated (e.g., only some patients were biopsied, PsO anamnesis was not reported for all case reports, etc.).

## 5. Conclusions

In conclusion, in our review we found two groups of patients who developed PsO during the treatment of dupilumab for AD. It is very important to perform a careful personal and familiar anamnesis both before prescribing a treatment, to evaluate the risk of developing a PsO reaction, and in case of an outbreak of “de novo” PsO or a flare of PsO in AD patients, to appropriately manage the adverse reaction. Moreover, we propose that a study of cytokine expression and blood proteomic markers study should be performed to guide the selection of proper biologic treatment. 

## Figures and Tables

**Table 5:** Features of histology of “de novo” PsO and relapse PsO.

Histology Features	“De Novo” PsO in Adults (71/93)	“De Novo” PsO in Pediatric Patients (4/8)	Relapsed PsO in Adults (8/10)
Parakeratosis	22	4	1
Intracorneal microabscesses	16	0	1
Elimination of granular layer	11	3	1
Lymphocytic infiltrate in the upper dermis	11	1	0
Psoriasiform hyperplasia	6	2	2
Hyperkeratosis	8	2	0
Acanthosis	9	2	1
Infiltrates of macrophages or neutrophils in dermis	10	0	0
Spongiosis	10	0	1
Dilated capillaries	5	0	3
Thinning of supra-papillary plates	3	2	0
Exocytosis lymphocytes	5	0	0
Rete ridge elongation	3	1	0
Perivascular infiltrates	4	0	0

**Table 6:** Main findings from the reviewed and new cases.

	“De Novo” Pso in Adults (n = 94)	“De Novo” Pso in Pediatric Patients (n = 9)	Relapses of Pso in Adults (n = 11)	Relapses of Pso in Pediatric Patients (n = 1)
**Sex**				
M	58 (61.7)	7 (77.8)	4 (36.4)	0 (0)
F	36 (38.3)	2 (22.2)	7 (63.6)	1 (100)
**Age (average)**	45.8	13.6	27.5	9
**Time to psoriasis presentation (average months)**	5.6	6.4	4.8	4.8
**Type of psoriasis**				
Plaque	35 (68.6)	9 (100)	10 (90.9)	1 (100)
Pustular	8 (15.7)	1 (11.1)	0	0
Erythrodermic	3 (5.9)	0	3 (27.3)	0
Guttate	3 (5.9)	0	1 (9.1)	0
Inverse	3 (5.9)	0	0	0
Nummular	1 (1.9)	0	0	0
**Localization**				
Face and scalp	18 (41.9)	4 (44.4)	6 (54.5)	0
Trunk	14 (32.6)	4 (44.4)	4 (36.4)	0
Upper extremities	21 (48.8)	5 (55.6)	10 (90.9)	1 (100)
Lower extremities	22 (51.2)	6 (66.7)	5 (45.5)	0
**Histology**				
Parakeratosis	22 (30.9)	4 (100)	1 (12.5)	0
Intracorneal microabscesses	16 (22.5)	0	1 (12.5)	0
Elimination of granular layer	11 (15.5)	3 (75)	1 (12.5)	0
Lymphocytic infiltrate in the upper dermis	11 (15.5)	1 (25)	0	0
Psoriasiform hyperplasia	6 (8.5)	2 (50)	2 (25)	0
Hyperkeratosis	8 (11.3)	2 (50)	0	0
Acanthosis	9 (12.7)	2 (50)	1 (12.5)	0
Infiltrates of macrophages or neutrophils in dermis	10 (14.1)	0	0	0
Spongiosis	10 (14.1)	0	1 (12.5)	0
Dilated capillaries	5 (7.0)	0	3 (37.5)	0
Thinning of supra-papillary plates	3 (4.2)	2 (50)	0	0
Exocytosis lymphocytes	5 (7.0)	0	0	0
Rete ridge elongation	3 (4.2)	1 (25)	0	0
Perivascular infiltrates	4 (5.6)	0	0	0
Dupilumab interruption	37 (40.2)	4 (50)	4 (36.4)	0
Remission of Pso	64 (78.0)	9 (100)	4 (100)	1 (100)
**Management**				0
Topical steroids	54 (60.7)	8 (88.9)	5 (50.0)	1 (100)
NB-UVB	12 (13.5)	0	0	0
Topical calcipotriol and steroid	11 (12.4)	0	0	0
Methotrexate	5 (5.6)	0	2 (20.0)	0
Cyclosporin	6 (6.7)	2 (22.2)	0	0
Oral steroids	4 (4.5)	0	0	0
Tacrolimus	3 (3.4)	1 (11.1)	0	0
Pimecrolimus	0	0	1 (10.0)	0
Omalizumab	1 (1.1)	0	5 (50.0)	0
Mycophenolate mofetil	1 (1.1)	0	0	0
JAKi	5 (5.6)	2 (22.2)	0	0

The data in this table were available only in some of the reviewed cases. Please find below the number of cases with available data for each variable. Type of psoriasis: 51/94 in the “de novo Pso in adults” group, 9/9 in the “de novo Pso in pediatric patients” group, 11/11 in the “relapse of Pso in adults” group, 1/1 in the “relapse of Pso in pediatric patients” group. Localization: 43/94 in the “de novo Pso in adults” group, 9/9 in the “de novo Pso in pediatric patients” group, 11/11 in the “relapse of Pso in adults” group, 1/1 in the “relapse of Pso in pediatric patients” group. Histology: 71/94 in the “de novo Pso in adults” group, 4/9 in the “de novo Pso in pediatric patients” group, 8/11 in the “relapse of Pso in adults” group, 0/1 in the “relapse of Pso in pediatric patients” group. Dupilumab interruption: 92/94 in the “de novo Pso in adults” group, 8/9 in the “de novo Pso in pediatric patients” group, 10/11 in the “relapse of Pso in adults” group, 1/1 in the “relapse of Pso in pediatric patients” group. Remission of PsO: 82/94 in the “de novo Pso in adults” group, 9/9 in the “de novo Pso in pediatric patients” group, 4/11 in the “relapse of Pso in adults” group, 1/1 in the “relapse of Pso in pediatric patients” group. Management: 89/94 in the “de novo Pso in adults” group, 9/9 in the “de novo Pso in pediatric patients” group, 10/11 in the “relapse of Pso in adults” group, 1/1 in the “relapse of Pso in pediatric patients” group.

## Data Availability

Not applicable.
